# Age-Friendly Approach Is Necessary to Prevent Depopulation: Resident Architectural Designers and Constructors’ Evaluation of the Age-Friendliness of Japanese Municipalities

**DOI:** 10.3390/ijerph20176626

**Published:** 2023-08-22

**Authors:** Kazumasa Yamada, Kenta Murotani, Makiko Mano, Youngmi Lim, Jun Yoshimatsu

**Affiliations:** 1 Organization for Co-Creation Research and Contributions, Nagoya Institute of Technology, Gokiso-chou, Showa-ku, Nagoya 466-8555, Japan; 2 Liaison Office, Innovation Center for Translational Research, National Center for Geriatrics and Gerontology, 7-430, Morioka-chou 474-8511, Japan; 3 Biostatistics Center, Kurume University, 67, Asahi-machi, Kurume 830-0011, Japan; kmurotani@med.kurume-u.ac.jp; 4 College of Human Sciences, Kinjo Gakuin University, 2-1723, Omori, Moriyama-ku, Nagoya 463-8521, Japan; junyoshim@kinjo-u.ac.jp

**Keywords:** age-friendliness, aging society, rural aging, urban aging, demographic aging, depopulation, land price, single person household, outdoor spaces, Japan

## Abstract

Japan has the world’s largest old population ratio; thus, aging is an urgent societal issue. As global trends seem to be following Japan’s social changes, there is an emphasis on municipalities becoming more age-friendly. Hence, we examine the age-friendliness of 135 Japanese municipalities, selecting 240 resident architectural designers and constructors to assess their municipalities using the Age-Friendly Cities and Communities Questionnaire (AFCCQ). The findings indicate that Japan lacks “outdoor spaces and buildings”. Additionally, the evaluation of “housing”, “community support and health services”, and “transportation” in populated municipalities in the past five years was found to be significantly higher than that in depopulated ones. Age-friendliness is significantly affected by the AFCCQ total score (hereafter, Score) based on “housing”, “social participation”, “community support and health services”, “transportation”, and “financial situation” evaluations. High specificity (0.939) was found when the score was treated as a marker of depopulation; an age-friendly approach is a necessary condition for preventing depopulation. Furthermore, a lack of “communication and information” was observed in municipalities with a higher rate of single-person households aged 65 years and older. Therefore, resident architectural designers’ and constructors’ assessments, combined with the AFCCQ, will be a powerful tool for evaluating the age-friendliness of municipalities.

## 1. Introduction

According to the United Nations, in the 1990s, Japan became the world’s first super-aging society [1], plagued by low birth rates and high life expectancies [2]. Consequently, Japan’s population began to decline in 2008 [3]. Demographic aging is a global trend with significant implications for human development in the present century. The global life expectancy at birth was only 46.5 years in 1950; however, it exceeded 60 years in 1979, 65 years in 1996, and 70 years in 2010, respectively. The average global life expectancy in 2022 (predicted value) was 71.7 years; this is expected to exceed 75 years by 2033 and 80 years by 2077, respectively [4]. The Organization for Economic Co-operation and Development (OECD) reported that the number of people aged 65 years and older, as a proportion of the global population, was 5.1% in 1950 and is expected to reach 25.1% by 2050 in its member states [5]. This indicates that global trends seem to be following Japan’s social changes. Therefore, studying the country’s current situation may help to improve our understanding of the world’s aging society. The number of handrails installed in Japan increased more than threefold between 1993 and 2016 [2]. Accordingly, in response to Japan’s aging population, renovation work has been carried out for each house through measures such as public subsidies that promote barrier-free living. However, considering future developments, even if we aim to redevelop the entire city with an awareness of the flow of older people, there are issues pertaining to the interest in and ownership of existing facilities, and further research is required. Countries concerned about aging populations are now planning countermeasures. In the case of Japan, for an example, Obu City enacted ordinances that establish standards for road structure to promote smooth movement of elderly people and persons with disabilities in 2012 and a promotion ordinance relating not to anxiety about dementia in 2017 (first in the City of Japan) [6].

Plouffe and Kalache reported the efforts of the World Health Organization (WHO) in assisting and promoting cities to become more “age-friendly” [7] through the Global Age-Friendly Cities Guide: Checklist of Essential Features of Age-Friendly Cities [8]. Recently, Dikken et al. developed the Age-Friendly Cities and Communities Questionnaire (AFCCQ) [9], consisting of 23 questions that are psychometrically valid and measure the experiences and opinions of older residents regarding the age-friendliness of their residential localities. Additionally, as buildings constitute cities and towns, architectural designers and builders are obliged to take an interest in the living environment surrounding the buildings because of their occupational requirements. To investigate how people belonging to the occupations mentioned above evaluate age-friendly indicators in cities and towns in which they reside, we evaluated the age-friendliness of Japanese cities and towns built by resident architects and constructors, using them as subjects. The survey participants were expected to provide different evaluations depending on their involvement with their town. We wish to determine how architectural designers and constructors involved in urban development in Japan, where the population is aging and declining, evaluate the towns they live in from the perspective of “age-friendliness”. Therefore, we considered their responses to the survey from two perspectives: evaluation as a resident and evaluation as an expert.

## 2. Materials and Methods

From 1 August 2021 to 30 September 2022, e-mails explaining the research and Google Forms containing the survey questions were sent to architectural firms and design offices in Japan whose email addresses were publicly available. The subjects included people in town development-related occupations (architects, constructors, civil engineers, interior decoration designers, people involved in city planning, etc.), above 20 years of age, who could access and respond to information online through smartphones or personal computers during the above-mentioned period. The survey questionnaire, based on the AFCCQ, consisted of 23 questionnaire items in English juxtaposed with a Japanese translation, which the participants were asked to evaluate using a 5-scale classification of “Totally disagree” (−2), “Disagree” (−1), “Neutral” (±0), “Agree” (+1), and “Totally Agree” (+2).

The primary endpoint was the AFCCQ total score (hereafter, Score) consisting of 23 questionnaire items and 9 domains. The research subjects were selected based on their place of residence (up to the municipality), age, gender, educational history, period of living in the current location, place of residence five years ago, type of residence, number of household members, presence/absence of support/nursing care required, type of certification, presence/absence of chronic illness, use of a wheeled walker or wheelchair, etc. The nine domains, which we quoted from [9], included “public spaces and buildings”, “transportation”, “housing”, “social participation”, “respect and social inclusion”, “civic participation and employment”, “communication and information”, “community support and health services”, and “financial situation”. The “respect and social inclusion” were evaluated based on responses to two negative questions, and their values were reversed since the other 21 questions were positive. The data for each municipality were obtained from the 2020 National Census [10], and the public notice of January 2021 land prices [11]. In addition, data about “municipalities at risk of disappearing due to depopulation” were obtained from the report of the Japan Policy Council [12]. The statistical analysis software IBM SPSS Statistics version 27.0 was used to analyze the data, and Student’s *t*-test and Wilcoxon’s rank-sum test were performed to confirm the differences among residential municipalities. Contiguous quantities were summarized by mean, standard deviation, median, and interquartile ranges. Categorical variables were summarized by each segment, separated by groups of depopulated/populated municipalities in the past five years or the top 50/the worst 50 municipalities based on land price or nuclearization.

Additionally, a diagnostic performance plot (DP-plot) analysis [13] was created, showing the relationship between the score and sensitivity (SN), specificity (SP), and accuracy (AC). A receiver operating characteristic (ROC) curve analysis was also performed. To distinguish between depopulated (positive (+)) and populated (negative (−)) municipalities based on Score, the cut-off point was set to zero because Score = 0 represents “neutral” based on the definition of Score. Data management was conducted at the Nagoya Institute of Technology, and information managers, who were not directly involved in the study, were assigned the task of assigning arbitrary IDs to the data obtained from each target person. These managers confirmed their anonymity. Thereafter, the dataset was sent to the Biostatistics Center of Kurume University for statistical analysis. To fairly evaluate the results, we built and implemented a system in which people in charge of data management and statistical analysis were separated. In addition, we created a subject distribution map based on a blank map and location information obtained from 3kaku-K (https://www.freemap.jp/free.html (accessed on 21 August 2022)) and the Geospatial Information Authority of Japan (https://maps.gsi.go.jp/help/intro/school/blankmap.html#link04 (accessed on 21 August 2022)), respectively.

## 3. Results

Among the 240 participants who responded to the questionnaire, 199 (82.9%) were male and 41 (17.1%) were female. The sample consisted of 145 architects (60.4%), 46 constructors (19.2%), and 49 people in related occupations (20.2%), including those involved in city planning, interior design, and civil engineering. The average age (SD) was 57.3 (12.7) years. Furthermore, 163 subjects (77.9%) were <65 years old, and 77 subjects (32.1%) were ≥ 65 years old. Of all the participants, 83 (34.6%) had a chronic illness, and 36 (15.0%) had joint pain. In terms of their education level, 43 (17.9%) had passed graduate school, 153 had completed university (63.8%), 23 had completed junior college (9.6%), and 21 had completed senior high school (8.8%). The average number of household members (SD) was 2.9 (1.4), and the ratio of men to women in these households was 48.9%. Of all the participants, 98 (40.8%) had older people living with them in their households. 60 had one (25.0%), including 7 participants living alone (2.9%), 34 had two (14.2%), including 6 solitary older pairs (2.5%), and four had three (1.7%), including one solitary older trio (0.4%). Six subjects used a wheeled walker or wheelchair (2.5%), and 18 received care services in their household (7.5%). Based on care grades, these subjects were further categorized as follows: Four (1.7%) and five (2.1%) belonged to Yo Shien 1 and 2, respectively, and two (0.8%), two (0.8%), one (0.4%), four (1.7%), and zero (0%) belonged to Yo Kaigo 1, 2, 3, 4, and 5, respectively. These care-grade categories are defined below:

1. Yo Shien 1: Pre-classifying level 1: The person can perform most of the basic activities of daily living (ADL), including eating, excreting, moving, and bathing, on their own. However, to prevent the progression of symptoms, some support is required for instrumental activities of daily living (IADL), such as shopping, household chores, medication, and money management.
2. Yo Shien 2: Pre-classifying level 2: Compared with Yo Shien 1, the ability to perform IADL is reduced. Additionally, some assistance is required for personal care, and a person may require support when moving, e.g., standing up and walking.
3. Yo Kaigo 1: The ability to perform IADL is further reduced. The person now requires partial care; they may be unstable when standing or walking.
4. Yo Kaigo 2: In addition to Yo Kaigo 1, partial long-term care is required for ADL owing to forgetfulness and deterioration of comprehension.
5. Yo Kaigo 3: This condition requires full care in relation to ADL and IADL, as the person experiences reduced capacity to eat and bathe on their own.
6. Yo Kaigo 4: Compared with Yo Kaigo 3, the ability to move is further reduced, and the person finds it difficult even to excrete on their own, leading to difficulty in living their daily life without long-term care.
7. Yo Kaigo 5: The person’s ability to carry out both ADL and IADL is significantly reduced, requiring full assistance throughout their life. People at this stage may have difficulty communicating or may even be bedridden.

**Figure 1:**
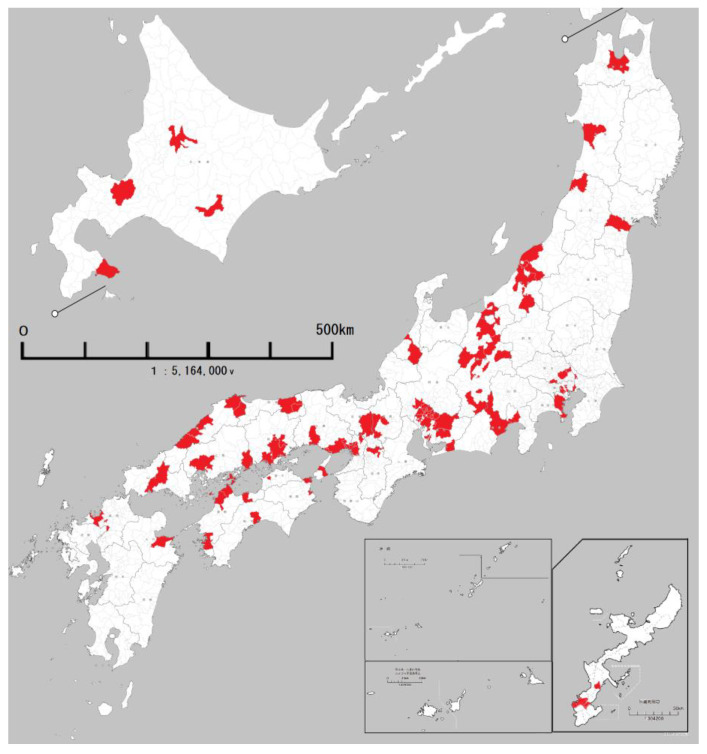
Distribution of the 135 municipalities in which the 240 subjects lived.

The subjects were spread across 135 municipalities in 35 prefectures, with the municipalities being distributed all over Japan, from Hokkaido in the north to Okinawa Prefecture in the south (Figure 1).

Table 1 summarizes the characteristics of the 135 municipalities. The municipalities consist of rural and urban populations with densities under 100 persons/km^2^ and over 10,000 persons/km^2^, respectively. In 109 municipalities (80.7%), the nuclear family ratio per household was greater than 50%. Additionally, in 125 municipalities (92.5%), the aging ratio was above 20% in both rural and urban areas. However, the fluctuation rate of households in Japan has increased over the past five years [9], with the number of households increasing in 119 municipalities (88.1%) and decreasing in 16 (11.9%). The trend of living alone has increased across all generations. Furthermore, isolation is a big problem in an aging society. Living with pets or moving is an important aspect of daily life. We found that 57 participants lived with their pets (23.6%). The dwelling period (SD) of the subjects was 22.9 (17.6) years on average; 196 (81.7%) had spent over five years in the same place, 29 (12.1%) had moved from another place, and 15 (6.3%) had moved within the same ward within a period of five years or less. Therefore, 211 subjects lived in the same ward or municipality.

Population fluctuations represent modern values, while land prices represent the future. Since the national census reflects an aging society, a survey of single-person households, aged 65 years and older, was conducted [10]. Therefore, we focused on the dynamic population change in the past five years, land in residential areas, and single households above 65 years of age, determining the ratios per total households in the aforementioned municipalities.

Table 2 shows that the average population and depopulation ratios were +3.09 ± 0.38% in 66 populated municipalities and −2.81 ± 0.27% in 69 depopulated municipalities during the past five years. Architectural designers and constructors negatively rated Japanese municipalities with regard to “outdoor spaces and buildings”. The “Score," "housing”, and “transportation” results for the 69 depopulated municipalities in the past five years were significantly lower than those of the 66 populated ones (*p* < 0.01). Similarly, the evaluation of “community support and health services” revealed significantly lower results in the 69 depopulated municipalities compared to the 66 populated ones (*p* < 0.05).

**Figure 2:**
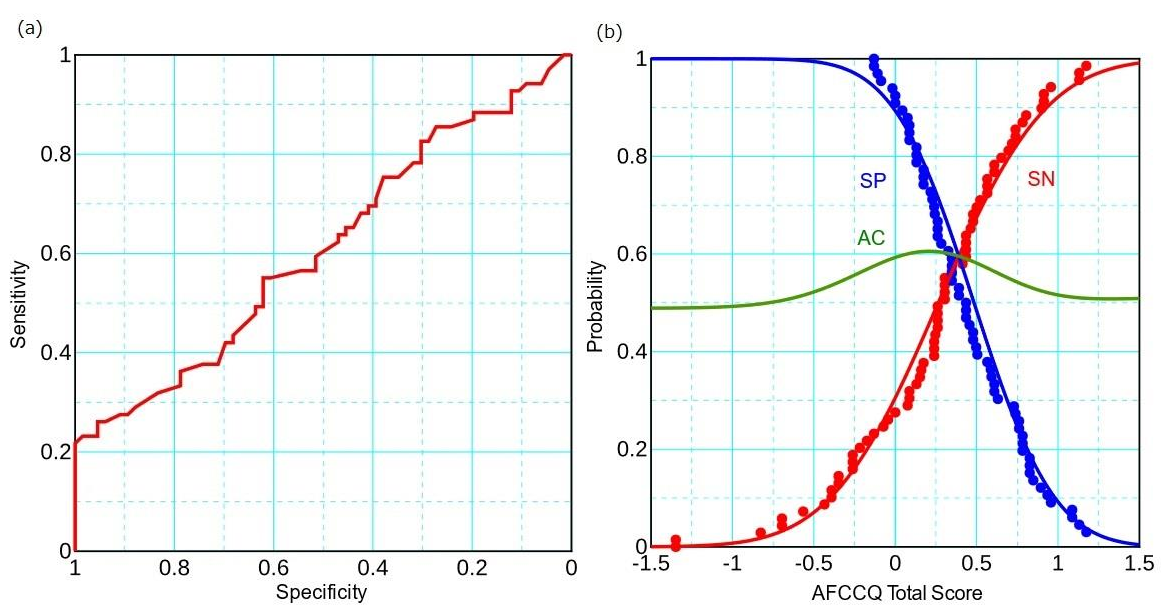
(**a**) ROC and (**b**) DP-plot analysis using Score as a marker for depopulation. SN (red), SP (blue), and AC (green) are logical curves.

We analyzed the discrimination threshold for depopulation using the AFCCQ total score as a marker (Figure 2).

The sensitivity (SN), the proportion of a Score ≥ 0 among populated municipalities, is 0.261; the specificity (SP), the proportion of a Score < 0 among depopulated municipalities, is 0.939. In the ROC analysis, the accuracy (AC) is 0.593, and the area under the curve (AUC) of the ROC curve is 0.609 (*p* = 0.029).

**Figure 3:**
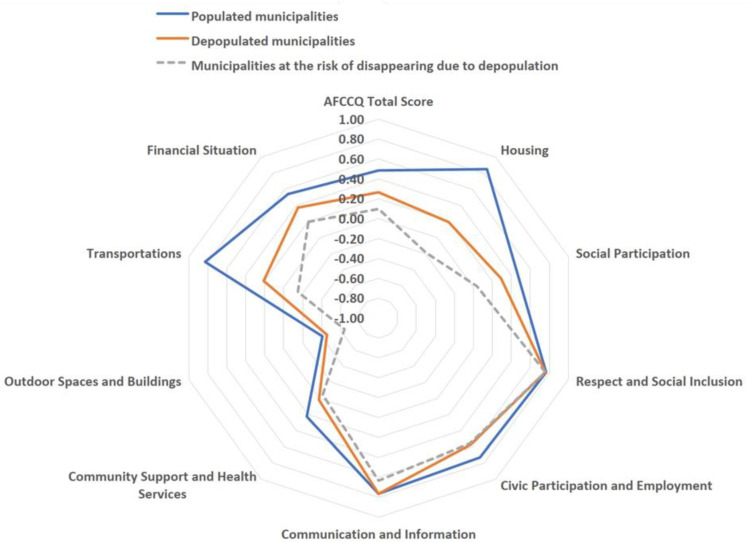
AFCCQ total score and 9 domains’ scores in populated/depopulated municipalities and those at risk of disappearing due to depopulation.

Japan has 1799 municipalities; however, the Japan Policy Council reported that by 2040, 896 municipalities would be at risk of disappearing due to depopulation as the number of resident women of childbearing age has halved in these municipalities [12]. Of the abovementioned municipalities, 19 are present in the 69 depopulated municipalities considered in this study, and they show lower evaluations in all aspects except “respect and social inclusion” (Figure 3). Each municipality was assessed by 1–9 resident architectural designers and constructors in Japan.

Table 3 shows the differences between the best and worst 50 municipalities with higher and lower land prices, respectively. The average unit price in the above-mentioned top 50 municipalities was 8.4 times higher than that in the worst 50 municipalities (*p* < 0.01). However, in the evaluation of “outdoor spaces and buildings”, the worst 50 municipalities with lower land prices were given negative evaluations in the index of “community support and health services” and “transportation”. The “Score," "housing," "transportation,” and “financial situation” of the higher land price municipalities were deemed to be significantly better than those of the lower land price municipalities (*p* < 0.01). Additionally, the “social participation” and “community support and health services” scores of the top 50 higher land price municipalities were significantly higher than those of the 50 worst lower land price ones (*p* < 0.05).

The national census, conducted on 1 October 2020, in Japan, reported a change in the ratio of single households consisting of a single person ≥65 years old per total households in each municipality during the past five years [10]. Table 4 shows the difference in the evaluation between the worst 50 municipalities with a lower ratio and the top 50 municipalities with a higher ratio of ≥65 years old, single households per total households.

The average ratio of the worst 50 municipalities with a lower ratio of ≥65 years for the single household per total households is 9.2 ± 0.2%, while that of the top 50 is 14.2 ± 0.3% (*p* < 0.01). Additionally, the evaluation of “communication and information” in the worst 50 municipalities with a lower ratio of ≥65 years for single households is significantly higher than that in the top 50 municipalities (*p* < 0.05), although the difference in the ratio is only 5% between the two groups.

## 4. Discussion

Residential architectural designers and constructors have a good understanding of “housing”, “outdoor spaces and buildings”, and “transportation” as professionals and a good understanding of other domains as residents. Thus, the score is a comprehensive evaluation supported by experience both as a professional and as a resident. We observed high (0.939) and low (0.261) sensitivities to depopulation using the Score in the ROC analysis, indicating that, although the age-friendly approach represented by the Score is a necessity, it is not sufficient to prevent depopulation. The design and arrangement of public green spaces promote health and well-being. Social relationships and an adequate environment are key challenges for increasingly population-dense cities. As cities become denser, public green spaces become more significant [14,15,16]. Outdoor and indoor environments influence older adults’ mobility, independence, and quality of life. Both the number and quality of public green spaces in the living environment are crucial [17]. In Japan, where the plains are narrow, setting up outdoor spaces poses difficulties, which is reflected in the low evaluation of “outdoor spaces and buildings." A declining population and an increased number of single-person households have appeared in Japan as a result. Japan has a highly convenient social structure that boasts material wealth, typified by the development of convenience stores. In pursuit of economic efficiency, Japan appears to have placed less emphasis on raising children, and the declining birth rate and an aging population have led to an increasing number of people of all generations living alone in Japan.

In the present century, demographic aging and urbanization have become global trends with significant implications for human development. Over the past two centuries, the development of various industries and improvements in social, environmental, and biological factors, including sanitation, housing, human engineering, and medical care, have led to an overall increase in longevity worldwide [18].

Dr. Albert Schweitzer stated that human ethics exist only when you dedicate yourself to helping those who are suffering [19]. However, these ethics are now being called into question. An aging society requires support from others, such as caregivers and helpers. Without opportunities to come into close contact with older people, there are no opportunities to help them. An increase in single-person households may indicate a critical situation, significantly influencing Japan’s future. When Mother Teresa came to Japan, she said, “In Japan, which seems to be rich, is not there a hunger in the heart? Being unwanted, unloved, uncared for, and forgotten by everybody, I think that is a much greater hunger, a much greater poverty than the person who has nothing to eat. Dear all in Japan, do not forget poverty, please”. We are entering an era in which Mother Teresa’s words are deeply resonating with people of all ages [20].

The WHO promotes age-friendliness in cities and communities worldwide by proposing policies, services, and structures for age-friendly cities and communities [21]. They are related to the physical and social environment and are designed to support older people, enabling them to have an active and healthy lifestyle. Therefore, it is necessary to assess the age-friendliness of cities and communities, using transparently constructed and validated surveys and measuring their structure in all respects. Qualitative approaches have been reported to measure and assess the age-friendliness of cities using photo-production [22], photo-voice methods [23], and citizen science research programs [17,24]. Various researchers have attempted to develop a more quantitative approach to measuring age-friendliness based on the Checklist of Essential Features of Age-Friendly Cities [21]. Luciano et al. [25] proposed a framework for assessing the age-appropriateness of housing using 71 metrics, summarized into eight domains, to detect and identify physical and non-physical features of a home environment that will enable residents to grow older in the same place.

Buildings constitute cities and towns. Due to their occupation, those involved in architectural design and construction become connected with people’s lives and, therefore, must take an interest in the living environment around the buildings. In this study, to survey age-friendliness in cities and towns in the best possible way, we combined the assessments of architectural designers and constructors in Japan with a survey based on the AFCCQ because, in addition to being transparent, reproducible, and having a compact volume of items, the AFCCQ is useful for identifying problems and their solutions in aging societies.

## 5. Conclusions

We surveyed 135 municipalities in Japan to gauge their age-friendliness. The results explored Japanese demographic aging, including urban and rural aging. Selecting 240 architectural designers and constructors in Japan, we conducted a survey based on the AFCCQ. The findings highlight that Japan lacks “outdoor spaces and buildings”, and a lack of “communication and information” was also observed in cities and towns with a higher rate of single-person households aged ≥65 years. Evaluation of “housing”, “community support and health services”, and “transportation” in populated municipalities in the past five years showed significantly better results than those in depopulated municipalities. Age-friendliness was significantly affected by land prices. According to the ROC curve analysis, an age-friendly approach, represented by the score, is necessary to prevent depopulation, making the AFCCQ a powerful tool for evaluating the age-friendliness of municipalities in combination with the assessment of resident architectural designers and constructors. The lack of an age-friendly approach is one of the reasons why demographic aging, including the extension of healthy life expectancy, and depopulation coexist.

## Figures and Tables

**Table 1:** Background data on the municipalities in which the subjects resided.

		Municipalities	Subjects
Number		135	240
Dynamic population during the past 5 years	Increased population	n = 66 (48.9%)	n = 135 (56.3%)
	Decreased population	n = 69 (51.1%)	n = 105 (43.8%)
	(Designated as the municipalities facing a risk of disappearing due to depopulation ^1^)	(n = 19 (14.1%))	(n = 26 (10.8%))
Population in each municipality			
	Under 10,000	n = 1 (0.7%)	n = 1 (0.4%)
	10,001–200,000	n = 83 (61.5%)	n = 139 (57.9%)
	200,001–400,000	n = 36 (26.7%)	n = 71 (29.6%)
	400,001–800,000	n = 13 (9.6%)	n = 28 (11.7%)
	800,001–1,600,000	n = 1 (0.7%)	n = 1 (0.4%)
Population density in each municipality			
	Under 100 persons/km^2^	n = 8 (5.9%)	n = 9 (3.8%)
	101–1000 persons/km^2^	n = 41 (30.4%)	n = 61 (25.4%)
	1001–10,000 persons/km^2^	n = 72 (53.3%)	n = 144 (60.0%)
	Over 10,000 persons/km^2^	n = 14 (10.4%)	n = 26 (10.8%)
Land price of residential areas in each municipality			
	Under 50,000 yen/m^2^	n = 42 (31.1%)	n = 54 (22.5%)
	50,000–100,000 yen/m^2^	n = 38 (28.1%)	n = 56 (23.3%)
	100,001–200,000 yen/m^2^	n = 31 (23.0%)	n = 75 (31.3%)
	200,001–800,000 yen/m^2^	n = 21 (15.6%)	n = 46 (19.2%)
	Over 800,000 yen/m^2^	n = 3 (2.2%)	n = 9 (6.7%)
Nuclear family ratio per total households			
	Under 50%	n = 26 (19.3%)	n = 71 (29.6%)
	at 50–60%	n = 74 (54.8%)	n = 117 (48.8%)
	Over 60%	n = 35 (25.9%)	n = 52 (21.7%)
Aging ratio ^2^			
	Under 20%	n = 10 (7.4%)	n = 15 (6.3%)
	at 20–30%	n = 82 (60.7%)	n = 167 (69.6%)
	at 30–40%	n = 42 (31.1%)	n = 57 (23.8%)
	Over 40%	n = 1 (0.7%)	n = 1 (0.4%)
Fluctuation rate of households in the past 5 years			
	Increased households under 5%	n = 62 (45.9%)	n = 102 (42.5%)
	at 5–10%	n = 47 (34.8%)	n = 86 (35.8%)
	over 10%	n = 10 (7.4%)	n = 22 (9.2%)
	Decreased households	n = 16 (11.9%)	n = 30 (12.5%)

^1^ Designated as the municipalities facing a risk of disappearance as a part of the “decreased population”. ^2^ Number of people ≥65 years old/total population.

**Table 2:** AFCCQ total score and 9 domains’ scores of populated and depopulated municipalities assessed by resident architectural designers and constructors in Japan.

AFCCQ Total Score and 9 Domains’ Scores	Populated Municipalities in the Past 5 Years	Depopulated Municipalities in the Past 5 Years	*p*-Value
Number of municipalities	66	69	
Population or Depopulation ratio (mean ± SE)	+3.09 ± 0.38%	−2.81 ± 0.27%	**<0.01 ^2^**
AFCCQ total score (mean ± SE)	0.48 ± 0.05	0.26 ± 0.06	**<0.01**
Domains of AFCCQ (mean ± SE)			
Housing	0.85 ± 0.05	0.19 ± 0.10	**<0.01**
Social participation	0.46 ± 0.07	0.29 ± 0.08	0.11
Respect and social inclusion	0.77 ± 0.09	0.76 ± 0.12	0.96
Civic participation and employment	0.73 ± 0.08	0.57 ± 0.10	0.22
Communication and information	0.76 ± 0.07	0.76 ± 0.10	1.00
Community support and health services	0.23 ± 0.07	0.02 ± 0.07	**<0.05**
Outdoor spaces and buildings	−0.41 ± 0.09 ^1^	−0.46 ± 0.11	0.73
Transportation	0.83 ± 0.12	0.21 ± 0.14	**<0.01**
Financial situation	0.54 ± 0.10	0.37 ± 0.10	0.23

^1^ Red-colored data show minus data. ^2^ Bold data show significant differences among two groups.

**Table 3:** Comparative study of AFCCQ total score and 9 domains’ scores between top 50 municipalities with higher land prices and worst 50 municipalities with lower land prices.

AFCCQ Total Score and 9 Domains’ Scores	Top 50 Municipalities with Higher Land Prices	Worst 50 Municipalities with Lower Land Prices	*p*-Value
Number of municipalities	50	50	
Average unit price (mean ± SE)	¥309,943 ± 45,250/m^2^	¥36,830 ± 2239/m^2^	**<0.01 ^2^**
AFCCQ total score (mean ± SE)	0.52 ± 0.06	0.19 ± 0.07	**<0.01**
Domains of AFCCQ (mean ± SE)			
Housing	0.87 ± 0.12	0.08 ± 0.15	**<0.01**
Social participation	0.46 ± 0.08	0.22 ± 0.09	**<0.05**
Respect and social inclusion	0.73 ± 0.13	0.80 ± 0.12	0.69
Civic participation and employment	0.68 ± 0.09	0.62 ± 0.12	0.64
Communication and information	0.71 ± 0.10	0.80 ± 0.10	0.53
Community support and health services	0.27 ± 0.08	−0.03 ± 0.09	**<0.05**
Outdoor spaces and buildings	−0.34 ± 0.12 ^1^	−0.60 ± 0.12	0.12
Transportation	1.04 ± 0.11	−0.08 ± 0.16	**<0.01**
Financial situation	0.65 ± 0.10	0.25 ± 0.12	**<0.01**

^1^ Red-colored data show minus data. ^2^ Bold data show significant differences among two groups.

**Table 4:** Comparative study of the AFCCQ total score and 9 domains’ scores between the worst 50 municipalities with a lower ratio and the top 50 municipalities with a higher ratio of ≥65 years, single household per total households.

AFCCQ Total Score and 9 Domains’ Scores	Worst 50 Municipalities with a Lower Ratio of ≥65 Years Old, Single Household per Total Households	Top 50 Municipalities with a Higher Ratio of ≥65 Years Old, Single Household per Total Households	*p*-Value
Number of municipalities	50	50	
Ratio of over-65-aged single-household per total households (mean ± SE)	9.2 ± 0.2%	14.2 ± 0.3%	**<0.01 ^2^**
AFCCQ total score (mean ± SE)	0.45 ± 0.04	0.36 ± 0.08	0.27
Domains of AFCCQ (mean ± SE)			
Housing	0.70 ± 0.11	0.44 ± 0.16	0.18
Social participation	0.44 ± 0.08	0.36 ± 0.10	0.54
Respect and social inclusion	0.90 ± 0.10	0.80 ± 0.12	0.52
Civic participation and employment	0.70 ± 0.09	0.62 ± 0.11	0.59
Communication and information	0.97 ± 0.08	0.66 ± 0.12	**<0.0** **5**
Community support and health services	0.14 ± 0.06	0.12 ± 0.10	0.83
Outdoor spaces and buildings	−0.41 ± 0.10 ^1^	−0.48 ± 0.13	0.68
Transportation	0.58 ± 0.14	0.54 ± 0.17	0.87
Financial situation	0.55 ± 0.10	0.49 ± 0.12	0.7

^1^ Red-colored data show minus data. ^2^ Bold data show significant differences among two groups.

## Data Availability

The datasets generated during and/or analyzed during the current study are not publicly available but are available from the corresponding author on reasonable request with the consent of all authors.
